# Biomarkers in ANCA-Associated Vasculitis-Associated Interstitial Lung Disease: A Scoping Review

**DOI:** 10.3390/biomedicines14102202

**Published:** 2026-09-29

**Authors:** Tommaso Pianigiani, Akter Dilroba, Michael McConville, Samantha Banford, Martina Meocci, Silvia Grazzini, Laura Bergantini, Ashley Elliott, Edoardo Conticini, Elena Bargagli, Nazia Chaudhuri, Paolo Cameli

**Affiliations:** 1 Respiratory Diseases Unit, Department of Medical Sciences, Surgery and Neurosciences University of Siena, 53100 Siena, Italy; tommaso.pianigian@student.unisi.it (T.P.);; 2 Respiratory Diseases Unit, Healthcare Provider for Interstitial Lung Diseases and Sarcoidosis, European Reference Network on Rare Respiratory Diseases (ERN-LUNG), Azienda Ospedaliera Universitaria Senese, 53100 Siena, Italy; 3 Belfast Health and Social Care Trust (BHSCT), Belfast BT12 6BA, UK; 4 Rheumatology Unit, Department of Medicine, Surgery and Neurosciences, University of Siena, 53100 Siena, Italy; 5 Centre for Experimental Medicine, School of Medicine, Dentistry and Biomedical Sciences, Queen’s University Belfast, Belfast BT9 7BL, UK; 6 School of Medicine, Ulster University, Northern Ireland UK Altnagelvin area Hospital, Westernhealth and Social Care Trust, Londonderry BT47 6SB, UK

**Keywords:** ANCA-associated vasculitis, interstitial lung disease, biomarkers

## Abstract

**Background:** Interstitial lung disease (ILD) is an increasingly recognized manifestation of ANCA-associated vasculitis (AAV) and is associated with substantial morbidity and mortality. The marked biological and clinical heterogeneity of AAV-associated ILD (AAV-ILD) poses major challenges for diagnosis, prognostic stratification, and therapeutic decision-making. Circulating biomarkers have emerged as promising tools for improving disease characterization; however, the available evidence remains limited and fragmented. **Methods:** We performed a scoping review to summarize the current evidence on circulating biomarkers investigated in AAV-ILD. Peer-reviewed studies evaluating biomarkers associated with ILD occurrence, disease activity, radiological progression, pulmonary function decline, mortality, or treatment response were systematically identified and qualitatively analyzed. **Results:** Seven observational studies met the inclusion criteria. The identified biomarkers could be broadly classified into three biological categories: epithelial injury biomarkers (KL-6 and SP-D), immune–fibrotic biomarkers (CCL2, CXCL6, CXCL13, and IL-13), and epithelial remodelling biomarkers (CA19-9, CA125, and CYFRA21-1). KL-6 and SP-D were associated across individual studies with pulmonary involvement, disease activity, or relapse, whereas CCL2, CXCL6, CXCL13, and IL-13 were associated with inflammatory and profibrotic processes, radiological abnormalities, or disease progression. Tumour-associated biomarkers were associated with radiological abnormalities and mortality, suggesting that they may reflect aberrant epithelial remodelling rather than malignancy. Across studies, circulating biomarkers showed preliminary associations with disease detection, prognosis, longitudinal disease behaviour, and treatment response; however, their clinical utility has not been prospectively established. Overall, the current evidence is limited by small sample sizes, retrospective study designs, and substantial clinical heterogeneity. **Conclusions:** Circulating biomarkers represent a promising approach for improving the biological characterization of AAV-ILD and may contribute to the future characterization of inflammatory and fibrotic disease phenotypes. Prospective multicentre studies with independent validation cohorts are required before biomarker-guided management can be implemented in routine clinical practice.

## 1. Introduction

ANCA-associated vasculitides (AAVs) are a group of systemic small-vessel disorders characterized by necrotising inflammation associated with antineutrophil cytoplasmic antibodies (ANCA), albeit with a variable prevalence of serum positivity [1]. The major clinicopathologic forms include granulomatosis with polyangiitis (GPA), microscopic polyangiitis (MPA), and eosinophilic granulomatosis with polyangiitis (EGPA) [1,2]. In these disease entities, ANCA are mainly directed against two specific antigens: proteinase-3 (PR3), primarily associated with GPA, and myeloperoxidase (MPO), more frequently found in MPA and EGPA [3]. Pulmonary involvement is common in AAVs with a wide spectrum of manifestations including diffuse alveolar haemorrhage, pulmonary nodules, airway disease, and interstitial lung disease (ILD) [4]. Among pulmonary manifestations, fibrotic ILD has emerged as a clinically relevant and increasingly recognised complication, particularly in patients with MPO-ANCA-positive MPA [5]. The relationship between AAV and ILD is complex and remains poorly understood. ILD may precede, coexist with, or follow the diagnosis of systemic vasculitis. A subset of patients initially diagnosed with idiopathic ILD may later develop ANCA positivity or clinically manifest vasculitis during follow-up [6]. Furthermore, isolated ANCA-positive ILD without clinical signs of systemic vasculitis has also been described and defined as ANCA-ILD [7].

The development and progression of AAV-ILD display marked heterogeneity. Some patients remain clinically stable for years, whereas others experience rapid fibrotic progression, recurrent exacerbations, respiratory failure, or death [4]. Radiologic patterns are also heterogeneous, ranging from usual interstitial pneumonia (UIP) to nonspecific interstitial pneumonia (NSIP), organizing pneumonia (OP), or mixed inflammatory-fibrotic phenotypes. This variability represents a major challenge for prognostic stratification and therapeutic decision-making [8]. Current management strategies for AAV-ILD remain largely empirical. Immunosuppressive agents such as glucocorticoids, cyclophosphamide, rituximab, mycophenolate mofetil, or azathioprine are generally employed based on evidence for other systemic manifestations of the disease, whereas antifibrotic agents have emerged as potential therapeutic options for progressive fibrotic forms [9,10]. However, no validated algorithms currently exist to guide treatment selection, timing, ormonitoring in AAV-ILD. In this setting, biomarkers have gained increasing attention as potential tools for improving disease characterisation, prognostic assessment, and therapeutic stratification, particularly circulating biomarkers, like Krebs von den Lungen-6 (KL-6), C-C motif ligand 2 (CCL2/MCP-1), surfactant proteins (SP), and serum oncomarkers such as carbohydrate antigen 19-9 (CA19-9), cancer antigen 125 (CA125) and cytokeratin-19 fragment (CYFRA21-1). The objective of this scoping review is to map and summarise the currently available evidence regarding biomarkers in AAV-ILD, focusing on their potential role in disease detection, prognostic assessment, progression monitoring, and treatment-response assessment.

## 2. Materials and Methods

A systematic search was performed in PubMed/MEDLINE, Scopus, and Web of Science to identify studies investigating biomarkers in patients with AAV-associated ILD. Studies published up to 20 September 2026 were considered. The literature search was last updated on 20 September 2026.

Only studies published in English were included. The search strategy combined terms related to ANCA-associated vasculitis, interstitial lung disease, and biomarkers. The following search string was used: (“ANCA-associated vasculitis” OR AAV OR microscopic polyangiitis OR MPA OR granulomatosis with polyangiitis OR GPA OR MPO-ANCA OR PR3-ANCA) AND (“interstitial lung disease” OR ILD OR pulmonary fibrosis OR interstitial pneumonia) AND (biomarker OR biomarkers OR KL-6 OR Krebs von den Lungen-6 OR CCL2 OR MCP-1 OR CXCL6 OR CA19-9 OR CYFRA21-1 OR CA-125 OR surfactant protein OR SP-A OR SP-D). The complete database-specific Boolean search strings, search fields, and applied restrictions are provided in Supplementary Table S1. Additional studies were identified through hand-searching and cross-referencing the bibliographies of the retrieved articles to ensure literature saturation.

Peer-reviewed studies investigating biomarkers associated with ILD occurrence, disease activity, radiologic progression, pulmonary functional decline, mortality, or treatment response in AAV were considered eligible. For each outcome domain, all relevant results reported in the included studies were considered, irrespective of the specific measure, time point, or statistical analysis used.

Conference abstracts, preprints, unpublished material, case reports, reviews, editorials, and grey literature were excluded. Two authors independently screened titles and abstracts for eligibility. Potentially relevant full-text articles were subsequently independently assessed by the same two reviewers for final inclusion. Discrepancies at either stage were resolved by discussion, and when consensus could not be reached, a third author adjudicated. No automation tools were used during the study selection process. Data extracted from the included studies comprised study design, study population and setting, ANCA subtype, investigated biomarkers, pulmonary and extrapulmonary involvement, and the main clinical outcomes and biomarker associations. Data extraction was performed by one reviewer and independently verified by a second reviewer, with discrepancies resolved by consensus. Data were charted in a standardized table and subsequently synthesized narratively according to biomarker category and clinical outcome. No assumptions or imputations were made for missing or unclear information; data were extracted as reported in the original publications. No predefined effect measure was selected because no quantitative synthesis or meta-analysis was performed; effect estimates were reported descriptively as provided by the individual studies.

Given the heterogeneity of the available evidence, including differences in study design, patient populations, radiologic patterns, evaluated biomarkers, and clinical outcomes, a scoping review methodology was preferred over a formal systematic review. The review was reported in accordance with the Preferred Reporting Items for Systematic Reviews and Meta-Analyses (PRISMA) 2020 statement. The review was not registered, and a formal review protocol was not prepared.

Given the exploratory nature of this scoping review and the heterogeneity of the available evidence, a formal risk-of-bias assessment was not performed. This work was conducted within the framework of the European Reference Network on Rare Respiratory Diseases (ERN-LUNG).

## 3. Results

A total of 19 records were initially identified through database searching across PubMed/MEDLINE, Scopus, and Web of Science. After removal of 2 duplicate records, 17 studies underwent title and abstract screening. Of these, 10 studies were excluded because of unsuitable study design, including case reports and review articles. Finally, 7 studies were included in the qualitative analysis, comprising 5 retrospective observational studies and 2 prospective observational studies, as depicted in Figure 1.

Most studies (5/7, 71.4%) consisted of retrospective observational cohorts involving relatively small patient populations, predominantly including patients with MPA and MPO-ANCA positivity. The analysed biomarkers have mainly been investigated in relation to the presence of ILD, disease activity, radiologic progression, pulmonary functional decline, treatment response, and mortality (Table 1). Given the observational nature of the available evidence, the heterogeneity in study design, sample size, biomarker assessment, and evaluated outcomes, and the absence of independent validation for most reported associations, the findings summarized below should be regarded as preliminary and hypothesis-generating rather than as evidence of validated diagnostic or prognostic performance.

Notably, all eligible studies evaluated biomarkers in peripheral blood, whereas no eligible studies assessing biomarkers in bronchoalveolar lavage fluid (BALF) or sputum in AAV-ILD were identified. The circulating biomarkers identified in the included studies can be broadly classified into three pathobiological categories according to their presumed cellular origin and biological role: epithelial injury biomarkers, immune–fibrotic biomarkers, and epithelial remodelling biomarkers. Figure 2 summarizes their proposed sources and pathogenetic pathways.

### 3.1. Epithelial-Derived Biomarkers

Among the available biomarkers, Krebs von den Lungen-6 (KL-6) is the most extensively studied molecule in AAV-ILD. KL-6 is a mucin-like glycoprotein expressed by injured or regenerating type II pneumocytes and is involved in epithelial proliferation and repair mechanisms [11]. In two small monocentric studies, including a retrospective cohort of 13 MPO-AAV patients (6 with ILD) and a prospective cohort of 13 AAV patients (4 with ILD), serum KL-6 levels were significantly higher in patients with AAV-ILD than in those without pulmonary involvement [12,13]. Although high diagnostic performance was reported in one of these studies, this estimate was derived from a very small cohort and from only a limited number of patients with ILD. Therefore, the reported diagnostic performance should be interpreted cautiously and requires external validation in larger, independent cohorts before any clinical application can be considered.

In the study by Iwata et al., serial KL-6 concentrations reflected the clinical course of interstitial pneumonia, decreasing during treatment-induced remission and increasing with active pulmonary involvement, suggesting a potential role in disease monitoring and treatment response assessment [12].

Surfactant proteins, particularly surfactant protein D (SP-D), have also been investigated in AAV-ILD. In the multicentre retrospective REVEAL cohort, including 134 patients with MPA-associated ILD, elevated baseline SP-D levels were associated with subsequent ILD relapse, defined as the recurrence of respiratory clinical signs and symptoms together with new interstitial shadowing on high-resolution computed tomography (HRCT). Together with KL-6 and the UIP pattern, SP-D contributed to risk stratification models for ILD relapse. Receiver operating characteristic (ROC) analysis identified optimal cut-off values of 430 U/mL for KL-6 (AUC 0.71) and 89.5 ng/mL for SP-D (AUC 0.75) for predicting ILD relapse. Higher relapse rates were in turn associated with increased all-cause and respiratory-related mortality. These findings suggest that surfactant proteins may reflect ongoing alveolar epithelial injury and could potentially contribute to prognostic assessment in AAV-ILD [14].

In a separate single-centre biomarker-profiling study, Matsuda et al. identified two phenotypic clusters among patients with MPA-ILD. Patients classified as clinically fibrotic-dominant showed higher KL-6 and SP-D levels than those with a clinically inflammatory-dominant phenotype, although these findings were exploratory and derived from a small subgroup analysis [15].

### 3.2. Inflammatory and Immune–Fibrotic Biomarkers

Beyond biomarkers of epithelial injury, additional mediators associated with inflammatory and profibrotic pathways have also been investigated in AAV-ILD.

In a monocentric retrospective study including 49 patients with MPA, of whom 32 had ILD, serum C-C motif chemokine ligand 2 (CCL2), also known as monocyte chemoattractant protein-1 (MCP-1), levels were significantly higher in patients with MPA-ILD than in those without ILD and remained associated with the presence of MPA-ILD after adjustment for age, sex, and elevated serum creatinine. Baseline CCL2 did not correlate with baseline pulmonary function or HRCT ground glass opacity (GGO) and fibrosis scores. However, higher baseline CCL2 concentrations were associated with a greater subsequent increase in HRCT fibrosis score over one year (r = 0.51, *p* = 0.005). Immunohistochemical analysis further demonstrated intense CCL2 expression in CD68+/CD163+ alveolar macrophages and metaplastic epithelial cells, supporting a potential role for macrophage-mediated inflammatory and profibrotic pathways in disease progression [16]. In a subsequent study, Matsuda et al. investigated a broader panel of 21 serum biomarkers in 49 patients with MPA, including 32 with ILD and 17 without ILD, together with 10 healthy controls. Biomarker profiling suggested a predominant Th2- and M2 macrophage-oriented immune phenotype in MPA-ILD. Among individual biomarkers, baseline serum CXCL13 levels correlated with the extent of GGO on HRCT (r = 0.53), whereas CXCL13 and IL-13 correlated with fibrosis scores (r = 0.38 and r = 0.35, respectively). Principal component and cluster analyses further identified two biomarker-defined MPA-ILD phenotypes, classified as clinically fibrotic-dominant and clinically inflammatory-dominant. The fibrotic-dominant group showed higher KL-6 and SP-D levels and a higher incidence of severe infections during follow-up (52.9% vs. 13.3%, *p* = 0.03). These findings suggest that multidimensional serum biomarker profiling may capture distinct inflammatory and fibrotic phenotypes within MPA-ILD, although these exploratory findings require validation in larger independent cohorts [15].

More recently, in a monocentric prospective study including 292 patients with AAV (132 with ILD) and 82 healthy controls, C-X-C motif chemokine ligand 6 (CXCL6) emerged as a potential biomarker of AAV-ILD. In this large cohort, serum CXCL6 levels were significantly higher in AAV-ILD compared with AAV patients without ILD. ILD progression, defined as pulmonary function decline and/or radiological progression during follow-up, was independently associated with elevated baseline CXCL6 levels, suggesting a potential role in both disease activity assessment and prognostic stratification [17].

### 3.3. Tumor-Associated Biomarkers

Several serum oncomarkers, including carbohydrate antigen 19-9 (CA19-9), cancer antigen 125 (CA125), and cytokeratin-19 fragment (CYFRA21-1), have also been evaluated in MPO-AAV-ILD. Among them, CA19-9 demonstrated an association with the presence of ILD in MPO-AAV and showed high specificity in the investigated cohort, with a cut-off value within the recommended normal range. Moreover, CA19-9 correlated with HRCT fibrosis scores, whereas CYFRA21-1 correlated with ground-glass opacity scores. Both CA19-9 and CYFRA21-1 were independently associated with increased all-cause mortality. Despite their validation as tumour-associated markers, their elevation in ILD may reflect epithelial damage and aberrant tissue remodelling processes occurring in fibrotic lung disease [18].

### 3.4. Clinical Phenotypes, Confounders, and Biomarker Assessment Timing

The analysis of the included studies reveals a high degree of systemic complexity, with overlapping pulmonary and extra-pulmonary manifestations acting as potential clinical confounders. For instance, Matsuda et al. reported that a subset of their cohort presented with concurrent diffuse alveolar hemorrhage (DAH) or inflammatory infiltrates [16]. Similarly, Conticini et al. identified concomitant DAH and pulmonary nodules alongside multi-systemic involvement in their AAV-ILD population [13].

The relationship between circulating biomarkers and systemic disease activity also varied across the included studies. Li et al. found that serum CXCL6 levels correlated significantly with the Birmingham Vasculitis Activity Score (BVAS), as well as with acute-phase reactants such as ESR and CRP [17]. Conticini et al. also observed a direct positive correlation between KL-6 and BVAS [13].

Regarding potential confounding by renal involvement, Iwata et al. reported that serum KL-6 levels remained low despite severe renal dysfunction. In a representative patient, markedly increased creatinine levels and elevated MPO-ANCA titres were not accompanied by increased KL-6 concentrations, which rose only after the subsequent development of active interstitial pneumonia and pulmonary haemorrhage. In the same study, KL-6 levels decreased during clinical remission following glucocorticoid therapy [12].

Finally, the duration of disease and the timing of biomarker assessment differed substantially across the included studies. Li et al. measured CXCL6 at baseline or at the time of diagnosis, thereby including patients with short disease duration [17]. Similarly, Matsuda et al. enrolled subjects at the time of admission for remission induction therapy, frequently coinciding with the diagnosis of both ILD and systemic vasculitis [16]. In contrast, the cohort evaluated by Conticini et al. consisted of patients with more established disease who had already undergone long-term clinical follow-up before biomarker assessment [13].

The main characteristics of the included studies, including study design and population, investigated biomarkers, follow-up and outcome assessment, and principal quantitative findings, are summarized in Table 1.

**Table 1:** Study-level characteristics and main findings of studies investigating circulating biomarkers in ANCA-associated vasculitis-associated interstitial lung disease (AAV-ILD). The table summarizes study design and population, investigated biomarker(s), follow-up and outcome assessment, and the main quantitative findings, including effect estimates and 95% confidence intervals where reported. Where standardized follow-up intervals, predefined longitudinal outcomes, or effect estimates were not available, this is explicitly indicated.

Study	Design and Population	Biomarker(s)	Follow-Up/Outcome Assessment	Main Findings and Effect Estimates
Iwata et al., 2001 [12]	Single-centre observational study; 13 p-ANCA/MPO-ANCA-related vasculitis patients, including 6 with interstitial pneumonia; Japan	KL-6	No standardized follow-up; serial post-glucocorticoid measurements in 4 patients. Interstitial pneumonia assessed by clinical findings, chest imaging and PFTs	KL-6: 1533.7 ± 473.8 U/mL in interstitial pneumonia vs. 251.1 ± 46.6 without and 212.2 ± 24.9 in healthy controls (*p* < 0.05). KL-6 decreased in 3/4 serially assessed patients and increased in one patient with progressive fatal pulmonary disease. No effect estimate/CI reported
Matsuda et al., 2021 [16]	Single-centre observational study; 49 MPA patients (32 MPA-ILD, 17 without ILD) and 10 healthy controls; Japan	CCL2/MCP-1	1 year; paired samples in 21 patients. HRCT GGO/fibrosis scores and longitudinal radiological change assessed	CCL2 higher in MPA-ILD: 327.6 vs. 249.1 pg/mL (*p* = 0.0024); adjusted association remained significant (*p* = 0.0018; effect estimate/CI not reported). ROC AUC 0.77, sensitivity 88%, specificity 65%. Baseline CCL2 correlated with subsequent fibrosis-score increase (r = 0.51, *p* = 0.005)
Conticini et al., 2022 [13]	Multicentre observational study; 12 evaluable AAV patients (6 MPA, 6 GPA), including 4 with ILD; Italy/Spain	KL-6	Baseline and post-treatment assessment where available; no standardized interval. ILD assessed clinically and by HRCT	KL-6: 972.8 ± 398.5 vs. 305.4 ± 93.9 U/mL (*p* = 0.004). ROC AUC 1.00; cut-off 368 U/mL; sensitivity 100%, specificity 87.5%. Baseline KL-6 correlated with BVAS (r = 0.578, *p* = 0.044). Estimates based on only 4 ILD cases
Yang et al., 2022 [18]	Single-centre retrospective cohort; 169 newly diagnosed MPO-AAV patients (101 ILD, 68 without ILD); China	CA19-9, CA125, CYFRA21-1, CEA, SCC-Ag, NSE	Median follow-up 22.7 months in ILD and 30.3 months in non-ILD. HRCT, PFT/oxygenation and all-cause mortality assessed	ILD association: CA19-9 OR 24.345 (95% CI 3.025–51.254); CA125 OR 3.795 (1.694–8.501); CYFRA21-1 OR 2.994 (1.422–6.302). CA19-9 correlated with fibrosis score (r = 0.498); CYFRA21-1 with GGO score (r = 0.316). CA19-9 and CYFRA21-1 were independently associated with mortality
Matsuda et al., 2022 [15]	Single-centre observational study; 49 MPA patients (32 MPA-ILD, 17 without ILD) and 10 healthy controls; Japan	21 serum biomarkers; KL-6 and SP-D also evaluated	Pretreatment biomarker assessment; median 25.1 months for severe-infection outcomes. HRCT scores	CCL2 higher in MPA-ILD (*p* = 0.002); CXCL13 correlated with GGO (r = 0.53) and fibrosis (r = 0.38); IL-13 with fibrosis (r = 0.35). CFD vs. CID: higher KL-6 and SP-D.
Matsuda et al., 2024–REVEAL [14]	Multicentre retrospective cohort; 134 treated MPA-ILD patients from three Japanese centres	KL-6, SP-D	Mean 4.2 years; median 3.3 years (IQR 1.3–6.1). Relapse defined by recurrent respiratory signs/symptoms plus new HRCT interstitial abnormalities after remission induction, with alternative causes excluded	28/134 relapsed. KL-6: HR 1.0008/U/mL (95% CI 1.0002–1.001); SP-D: HR 1.006/ng/mL (1.003–1.009); UIP: HR 3.13 (1.08–9.05). ROC AUC 0.71 for KL-6 and 0.746 for SP-D. Relapse associated with all-cause mortality: HR 1.8 (1.04–3.22)
Li et al., 2025 [17]	Single-centre observational cohort; 292 AAV patients (132 AAV-ILD, 160 without ILD) and 82 healthy controls; China	CXCL6	Longitudinal clinical follow-up; mortality at 1 year. Progression defined as ≥ 5% relative FVC decline, ≥ 10% relative DLCO decline over ≥ 3–6 months, or radiological progression	Progression: 53.8% with high vs. 30.0% with low CXCL6 (*p* = 0.023). High baseline CXCL6 independently associated with progression: adjusted OR 3.207 (95% CI 1.238–8.312), *p* = 0.016. No association with 1-year mortality

Abbreviations: AAV, ANCA-associated vasculitis; ANCA, antineutrophil cytoplasmic antibody; AUC, area under the receiver operating characteristic curve; BVAS, Birmingham Vasculitis Activity Score; CA19-9, carbohydrate antigen 19-9; CA125, cancer antigen 125; CCL2, C-C motif chemokine ligand 2; CEA, carcinoembryonic antigen; CFD, clinically fibrotic-dominant; CI, confidence interval; CID, clinically inflammatory-dominant; CYFRA21-1, cytokeratin-19 fragment 21-1; DLCO, diffusing capacity of the lung for carbon monoxide; FVC, forced vital capacity; GGO, ground-glass opacity; GPA, granulomatosis with polyangiitis; HR, hazard ratio; HRCT, high-resolution computed tomography; ILD, interstitial lung disease; IQR, interquartile range; KL-6, Krebs von den Lungen-6; MCP-1, monocyte chemoattractant protein-1; MPA, microscopic polyangiitis; MPO-ANCA, myeloperoxidase-antineutrophil cytoplasmic antibody; NSE, neuron-specific enolase; OR, odds ratio; p-ANCA, perinuclear antineutrophil cytoplasmic antibody; PFT, pulmonary function test; ROC, receiver operating characteristic; SCC-Ag, squamous cell carcinoma antigen; SP-D, surfactant protein D; UIP, usual interstitial pneumonia.

A biomarker-centred synthesis of the available evidence, including the type of assessment (single or serial measurements), reported diagnostic and prognostic associations, and potential clinical applications, is provided in Table 2.

## 4. Discussion

This scoping review highlights the growing interest in circulating biomarkers as potential tools for the assessment and management of AAV-associated ILD. Given the wide biological and clinical heterogeneity of this condition, the identification of reliable indicators for disease activity and progression remains a major clinical priority. The variable disease trajectories observed in our review, which range from stable forms to rapidly progressive fibrotic phenotypes, underscore the urgent need for objective tools capable of improving prognostic stratification and potentially supporting clinical decision-making. However, none of the biomarkers identified in this review has yet demonstrated clinical utility for biomarker-guided therapeutic decision-making.

An important concept emerging from the available evidence is that the greatest unmet need in AAV-ILD is not simply the identification of biomarkers reflecting systemic vasculitis activity, but rather biomarkers specifically capturing pulmonary epithelial injury and fibrotic remodelling. Since AAV is a multisystem disease, however, the organ specificity of circulating biomarkers represents a major interpretative challenge. Their concentrations may reflect systemic inflammatory activity, extrapulmonary organ involvement, altered clearance, or comorbid conditions rather than pulmonary injury alone. This limitation differs among biomarker classes and should be considered when interpreting their associations with AAV-ILD. Biomarkers predominantly reflecting pulmonary epithelial injury may therefore provide complementary information on lung involvement, although their measurement in peripheral blood does not necessarily confer lung specificity.

Among the analysed biomarkers, KL-6 emerged as the most extensively investigated molecule. The association between elevated KL-6 levels and pulmonary involvement supports the hypothesis that epithelial injury and regeneration represent central mechanisms in AAV-ILD pathogenesis. Furthermore, the observed reduction in KL-6 levels following glucocorticoid therapy suggests a possible role in monitoring disease activity and treatment response.

Evidence derived from other ILD settings further reinforces the potential clinical relevance of KL-6 in AAV-ILD, while also highlighting its limited disease specificity. KL-6 has been extensively investigated in IPF and other fibrotic ILDs, where elevated levels have been associated with disease activity, progression and adverse outcomes [19,20,21]. Dynamic changes in KL-6 concentrations have also been investigated in relation to treatment response in different fibrotic lung diseases, supporting its potential value as a longitudinal monitoring biomarker [22]. Importantly, similar findings have been reported in autoimmune-associated ILDs. A meta-analysis including 29 studies showed that KL-6 discriminated CTD patients with ILD from those without ILD, with a pooled sensitivity of 0.76 and specificity of 0.89. However, the available evidence was insufficient to establish its ability to discriminate among individual CTD-ILD subtypes [23]. More recent meta-analytic evidence across fibrotic ILDs has also shown lower diagnostic performance of KL-6 in autoimmune-associated ILDs than in idiopathic interstitial pneumonias, further emphasizing the influence of disease context on its performance [24].

Although less extensively studied, surfactant proteins, particularly SP-D, have also emerged as potentially relevant biomarkers in AAV-ILD. Similar to KL-6, SP-D has been widely investigated in IPF and other fibrotic and inflammatory lung diseases, where it has been associated with alveolar epithelial injury and disease behaviour [25]. SP-D has also been extensively investigated in autoimmune-associated ILDs. The same meta-analysis of 29 CTD studies showed that SP-D discriminated CTD-ILD from CTD without ILD, with pooled sensitivity and specificity of 0.65 and 0.88, respectively, although KL-6 showed superior overall diagnostic performance [23]. Thus, rather than identifying a specific ILD aetiology, KL-6 and SP-D appear primarily to reflect alveolar epithelial injury shared across idiopathic and autoimmune-associated fibrotic lung diseases. Neither KL-6 nor SP-D is specific for AAV-ILD. KL-6/MUC1 may also be elevated in malignancies, particularly lung adenocarcinoma, whereas altered circulating SP-D levels have been reported in several non-ILD pulmonary diseases [26,27]. These biomarkers should therefore be interpreted primarily as indicators of alveolar epithelial injury rather than as disease-specific markers of AAV-ILD. Their potential value in AAV-ILD may lie in providing complementary information on pulmonary involvement and disease evolution, although prospective validation remains necessary.

While the available evidence in AAV-ILD is based on circulating measurements, assessment of biomarkers directly within the respiratory compartment may provide complementary biological information. KL-6 has been detected in bronchoalveolar lavage fluid (BALF) across different ILDs, where its concentrations have been associated with alveolar epithelial injury and circulating KL-6 levels [28]. More recently, paired BALF and serum measurements of KL-6 and SP-D have been investigated in ILD populations, suggesting that their compartmental distribution may differ according to the presence of pulmonary fibrosis [14]. However, these findings derive from non-AAV ILD populations, and we did not identify studies specifically evaluating KL-6 or SP-D in BALF or sputum in AAV-ILD. Whether respiratory-compartment measurements provide greater pulmonary specificity or additional prognostic information compared with circulating biomarkers in AAV-ILD therefore remains unknown and warrants dedicated investigation.

CCL2 and CXCL6 may represent another biologically relevant group of biomarkers in AAV-ILD, as both chemokines are involved in immune cell recruitment, inflammatory amplification, and fibrotic remodelling. CCL2 primarily mediates monocyte and macrophage recruitment through CCR2 signalling, whereas CXCL6 promotes neutrophil recruitment and angiogenesis through CXCR1/2 receptors [29,30].

Beyond their inflammatory role, both molecules appear to contribute to epithelial–mesenchymal transition and to the immune–fibrotic crosstalk underlying progressive pulmonary fibrosis.

Evidence from other ILDs suggests that these chemokine pathways are not restricted to AAV-ILD. In IPF, CCL2 and related chemokine pathways have been implicated in monocyte/macrophage recruitment and profibrotic signalling [31]. CXCL6 provides an additional relevant link with IPF: increased pulmonary CXCL6 levels have been associated with disease progression and mortality, while experimental evidence indicates that CXCL6 promotes fibroblast collagen synthesis through CXCR1/2 signalling [32]. Similar findings have also been reported in autoimmune-associated ILDs. In systemic sclerosis, higher circulating CCL2 levels predicted a faster subsequent decline in FVC and poorer survival in two independent cohorts [33]. These observations indicate that CCL2- and CXCL6-related pathways are shared across idiopathic and autoimmune-associated fibrotic lung diseases rather than being specific to AAV-ILD. Accordingly, their potential value in AAV-ILD may lie in reflecting immune–fibrotic activity rather than in distinguishing AAV-ILD from other autoimmune-associated ILDs. Their limited organ specificity is particularly relevant in AAV. CCL2 is a systemic inflammatory chemokine implicated in several autoimmune, renal, cardiovascular, and neoplastic conditions, including inflammatory kidney disease [34]. Similarly, CXCL6 participates broadly in neutrophil recruitment, inflammation, tissue repair, and cancer [35]. Notably, in the AAV cohort included in this review, circulating CXCL6 also correlated with BVAS, ESR, and CRP, indicating that its circulating levels may partly reflect systemic vasculitic activity rather than pulmonary involvement alone.

Taken together, these observations suggest that CCL2 and CXCL6 may not only reflect ongoing inflammatory activity but also represent markers of the complex interaction between immune dysregulation, epithelial injury, and fibrotic remodelling characterising AAV-ILD. Their potential role as candidate biomarkers associated with pulmonary involvement and disease evolution warrants further investigation in a disease in which timely identification of progressive pulmonary involvement remains a major unmet clinical need.

The available evidence regarding serum oncomarkers further supports the concept that epithelial injury and aberrant tissue remodelling represent key components of pulmonary involvement in AAV-ILD. Although originally developed as tumour-associated markers, molecules such as CA19-9, CA125, CYFRA21-1, and CEA are increasingly recognised as markers of epithelial activation and regenerative abnormalities occurring in fibrotic lung diseases [36]. Collectively, these observations support the concept that tumour-associated biomarkers capture epithelial remodelling processes occurring during pulmonary fibrosis and may therefore complement conventional biomarkers of epithelial injury in the biological characterisation of AAV-ILD.

These findings appear consistent with the broader ILD literature. In a large retrospective cohort including both IPF and non-IPF ILDs, Kwon et al. demonstrated significantly higher serum levels of CEA, CA19-9, and CA125 in ILD patients compared with matched healthy controls, despite median values often remaining within normal laboratory ranges. Importantly, elevated CEA levels were independently associated with mortality, particularly in non-IPF fibrotic ILDs. The authors proposed that these biomarkers may reflect epithelial proliferation and bronchiolar/alveolar remodelling associated with ongoing fibrotic injury [37]. These observations are further supported by findings from the PROFILE study, one of the largest prospective biomarker cohorts in IPF. In this multicentre longitudinal study, epithelial-derived tumour-associated biomarkers such as CA19-9 and CA125 were associated with disease progression and mortality. Histopathological analyses further demonstrated marked expression of these markers within metaplastic epithelial cells in fibrotic lung tissue, supporting the hypothesis that they reflect ongoing epithelial dysfunction and aberrant repair processes rather than occult malignancy [38].

Importantly, similar abnormalities have also been described in autoimmune-associated ILDs. In RA-ILD, serum CA19-9, CA125, and CEA were significantly higher than in RA without ILD, with CA19-9 independently associated with the presence of ILD [39]. A subsequent systematic review and meta-analysis confirmed significantly higher CEA and CA19-9 levels in RA-ILD than in RA without ILD, whereas the pooled difference in CA125 was not statistically significant [40]. These findings indicate that tumour-associated epithelial biomarkers are not specific to AAV-ILD and show substantial overlap across autoimmune-associated and idiopathic fibrotic ILDs, suggesting that they predominantly reflect shared processes of epithelial injury and aberrant repair rather than the underlying disease aetiology. More broadly, tumour-associated markers have been associated with disease severity, pulmonary function decline, and survival across different fibrotic lung diseases [41].

The limited organ specificity of tumour-associated biomarkers is particularly important when interpreting their elevation in AAV-ILD. CA19-9 may increase in several non-malignant pancreatobiliary, hepatic, metabolic, and pulmonary conditions [42], whereas CA125 may rise in association with pleural or pericardial effusions, ascites, cirrhosis, and heart failure [43]. CYFRA21-1 is particularly susceptible to confounding by renal dysfunction, with circulating concentrations increasing as glomerular filtration declines [44]. This is especially relevant in AAV, in which renal involvement is frequent, and indicates that elevations of these markers cannot be attributed to pulmonary epithelial remodelling without considering concomitant systemic manifestations and comorbidities.

Taken together, these data support the hypothesis that serum oncomarkers may act as indirect indicators of epithelial dysfunction and aberrant repair mechanisms in AAV-ILD. Their reported associations with ILD, radiological abnormalities, and mortality support further investigation of their potential role in the phenotypic characterisation of pulmonary involvement [41]. An additional aspect emerging from the available evidence is the relationship between circulating biomarkers and specific HRCT abnormalities. In the study evaluating tumour-associated biomarkers, CA19-9 correlated with fibrosis scores, whereas CYFRA21-1 correlated with ground-glass opacity scores. These associations may reflect, at least in part, different biological processes, with CA19-9 potentially capturing chronic epithelial remodelling and CYFRA21-1, a fragment released during epithelial injury and turnover, more closely reflecting active epithelial damage. Similarly, CXCL13 correlated with both ground-glass opacity and fibrosis scores, whereas IL-13 correlated with fibrosis scores, suggesting that immune-cell recruitment and Th2-related pathways may accompany different components of pulmonary injury [15]. In addition, higher baseline CCL2 concentrations were associated with a subsequent increase in HRCT fibrosis score during follow-up [16], consistent with its proposed role in monocyte/macrophage recruitment and profibrotic signalling.

These associations should, however, be regarded as exploratory and should not be interpreted as evidence of true biomarker specificity for individual HRCT patterns. Ground-glass opacity is a nonspecific radiological finding that may reflect inflammation, acute epithelial injury, alveolar haemorrhage, or fine fibrosis [45]. Accordingly, the observed biomarker–HRCT relationships may provide biological clues regarding the processes underlying pulmonary abnormalities but require confirmation in larger cohorts with standardized imaging assessment.

More broadly, comparison with IPF and autoimmune-associated ILDs highlights an important limitation regarding disease specificity. The substantial overlap of the biomarkers identified in AAV-ILD with those reported in other fibrotic lung diseases suggests that they predominantly capture shared biological processes rather than disease-specific molecular signatures. Current evidence therefore does not support their use to reliably distinguish AAV-ILD from other autoimmune-associated ILDs. Nevertheless, within an established clinical diagnosis, these biomarkers may provide complementary information on the biological processes underlying pulmonary involvement.

Molecules reflecting epithelial injury, inflammatory activation, immune–fibrotic crosstalk, and aberrant tissue remodelling appear capable of capturing different biological aspects of pulmonary involvement in AAV. Although the available evidence remains preliminary, the reported associations of several biomarkers with ILD occurrence, radiologic abnormalities, fibrosis progression, relapse, treatment response, and mortality support their further investigation as candidate markers for disease characterisation and longitudinal assessment. Their diagnostic and prognostic utility, however, remains to be established prospectively.

From a clinical perspective, the ultimate goal is not merely to identify the presence of ILD, but to distinguish the predominant biological process driving lung injury. Some biomarkers appear to reflect active epithelial damage and inflammatory pathways, whereas others seem to be more closely associated with chronic fibrotic remodelling. Although this concept still requires prospective validation, such biological phenotyping could potentially complement clinical, functional, and radiological assessment in defining individual disease phenotypes.

This aspect appears particularly relevant considering the substantial unmet clinical need in AAV-ILD. The coexistence of inflammatory and fibrotic mechanisms generates considerable uncertainty regarding disease classification, prognostic assessment, and therapeutic management. Inflammatory and fibrotic pulmonary phenotypes may require different therapeutic approaches, including immunosuppressive and antifibrotic strategies [46]; however, no included study prospectively evaluated biomarkers for treatment selection or demonstrated that biomarker-defined phenotypes can identify patients more likely to benefit from either therapeutic approach. Any potential biomarker-guided distinction between immunosuppressive and antifibrotic treatment should therefore currently be regarded as hypothesis-generating. Current treatment strategies remain largely empirical. Rituximab represents a key therapeutic option in GPA and MPA and has shown promising preliminary results in isolated AAV-ILD, particularly in combination with low-dose glucocorticoids [10]. Similarly, antifibrotic therapy with nintedanib represents a potential option in progressive fibrotic phenotypes [9], although evidence specifically addressing AAV-ILD remains scarce.

In this context, biomarkers might contribute to a more integrated assessment combining radiological, functional, serological, and clinical information. Their role in distinguishing inflammatory from predominantly fibrotic phenotypes is an attractive hypothesis but has not yet been prospectively validated in AAV-ILD. Moreover, biomarkers reflecting active alveolar injury or progressive fibrotic remodelling could potentially support, if prospectively validated, earlier identification of patients at risk of progression or poor response to therapy.

Another important unresolved issue concerns the integration of biomarkers with imaging assessment. HRCT currently represents the cornerstone for pulmonary assessment in AAV, but uncertainty persists regarding optimal screening and follow-up strategies, particularly in patients with isolated ANCA positivity or interstitial lung abnormalities (ILAs). The increasing recognition of ILAs and subclinical pulmonary fibrosis raises additional questions regarding the optimal timing of HRCT screening and longitudinal monitoring in AAV patients [46]. Biomarkers might, following appropriate prospective validation, help identify subjects who may warrant closer radiologic surveillance and more detailed clinical assessment.

Several limitations should be considered when interpreting the findings of this review. The available evidence is based on a small number of predominantly retrospective observational studies, with substantial heterogeneity in study design, sample size, patient populations, biomarker assessment, and evaluated clinical outcomes. Most reported biomarker associations have not undergone external validation, and no included study prospectively assessed biomarker-guided therapeutic decision-making. In addition, a formal methodological quality or risk-of-bias assessment was not performed, consistent with the exploratory scope of this review. A major interpretative challenge stems from the lack of phenotypic isolation; the frequent coexistence of acute alveolar injury, such as DAH, and chronic remodeling complicates the assessment of biomarker specificity for interstitial changes alone. Crucially, extra-pulmonary involvement introduces a confounding layer regarding whether these circulating signals reflect generic disease activity or organ-specific damage. In a systemic disorder like AAV, clinicians must be reasonably certain that fluctuating biomarker levels indicate localized pulmonary injury rather than a generalized inflammatory state or reduced renal clearance. This distinction is well illustrated by the contrast between markers: while specific chemokines track closely with broad systemic activity scores, serum KL-6 remained low despite severe renal dysfunction in one of the included studies and increased only after the development of active pulmonary disease [12], suggesting that it may be less susceptible to renal confounding than other circulating biomarkers. Conversely, markers such as CYFRA21-1 may be substantially influenced by impaired renal function, further emphasizing the need to interpret circulating biomarker levels in the context of the complete systemic phenotype.

The timing of biomarker assessment is a critical point, too. Early increases in biomarkers reflecting epithelial injury or immune activation might eventually contribute to the characterization of predominantly inflammatory phenotypes, whereas persistent elevations during later disease stages could potentially reflect established fibrotic remodelling. Although this hypothesis requires prospective confirmation, integrating biomarker trajectories with radiological and functional assessment could eventually contribute to a more refined longitudinal characterisation of pulmonary involvement and help determine whether biomarker changes reflect inflammatory activity or progressive fibrotic remodelling. However, further prospective validation and standardized cut-off values remain required before their routine implementation in clinical practice.

## 5. Conclusions

Biomarkers in AAV-ILD represent a promising field of investigation for improving the biological and clinical characterisation of this heterogeneous condition. Current evidence suggests that molecules such as KL-6, CCL2, SP-D, CA19-9, CYFRA21-1, and CXCL6 are associated with different aspects of pulmonary involvement, disease activity, progression, and prognosis. However, methodological limitations and the lack of prospective validation currently prevent their routine clinical implementation. Although encouraging, these observations remain hypothesis-generating and require prospective validation in larger multicentre cohorts before biomarker-guided management can be incorporated into routine clinical practice. Future research should focus on prospective multicentre studies incorporating both discovery and independent validation cohorts, with longitudinal integration of biomarker trajectories, radiological patterns, pulmonary function, and clinical outcomes. The development of validated biomarker panels may ultimately allow more accurate phenotypic characterisation and contribute to precision medicine approaches in AAV-associated ILD.

## Figures and Tables

**Figure 1:**
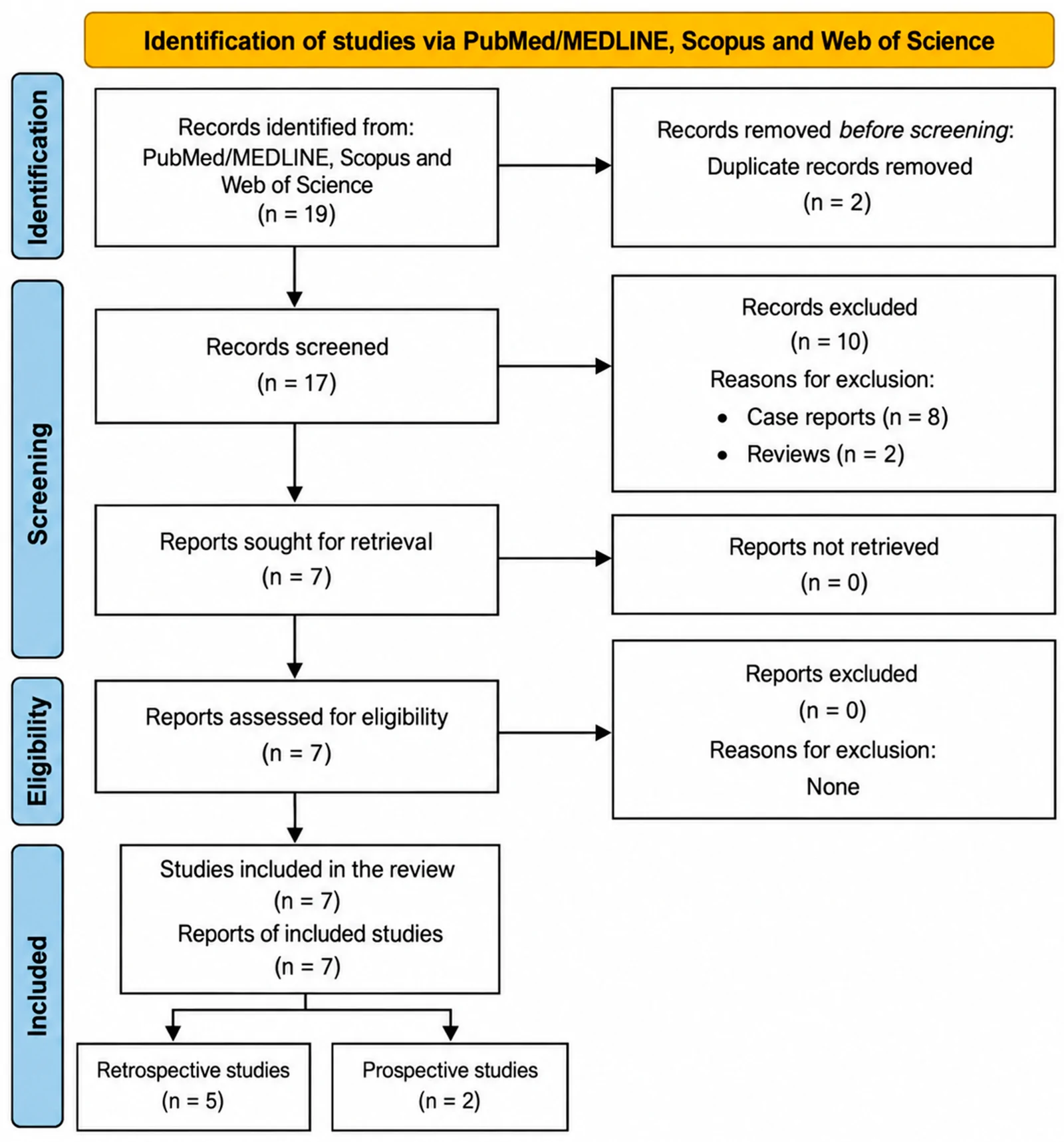
PRISMA 2020 flow diagram of the study selection process for the identification of studies investigating circulating biomarkers in ANCA-associated vasculitis-associated interstitial lung disease (AAV-ILD). After duplicate removal, title/abstract screening, and full-text assessment, 7 studies were included in the final qualitative synthesis.

**Figure 2:**
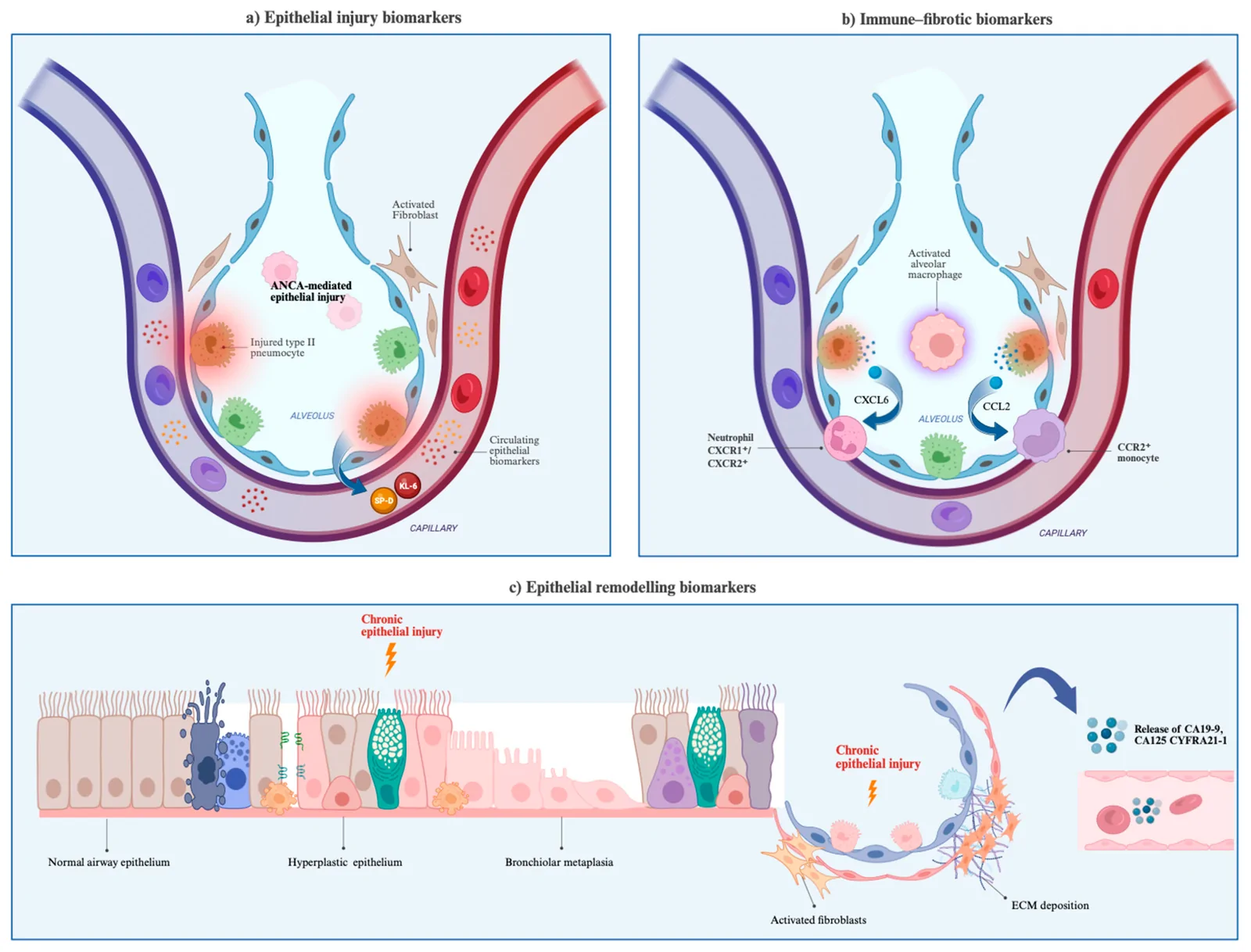
Conceptual framework illustrating the proposed biological origin and potential pathophysiological role of the principal circulating biomarkers investigated in ANCA-associated vasculitis-associated interstitial lung disease (AAV-ILD). The pathways illustrated are based on the known biological functions of these biomarkers and are intended as a conceptual representation; they were not directly demonstrated by the clinical studies included in this review. (**a**) Epithelial injury biomarkers. ANCA-mediated alveolar epithelial injury results in damage to type II pneumocytes, may lead to the release of epithelial-derived biomarkers, including KL-6 and surfactant protein-D (SP-D), into the circulation. (**b**) Immune–fibrotic biomarkers. Activated alveolar macrophages and injured epithelial cells produce chemokines involved in inflammatory cell recruitment. CCL2 promotes the recruitment of CCR2-positive monocytes/macrophages, whereas CXCL6 mediates neutrophil chemotaxis through CXCR1/CXCR2 signalling, potentially contributing to the amplification of inflammation and fibrogenesis. (**c**) Epithelial remodelling biomarkers. Chronic epithelial injury promotes epithelial hyperplasia, bronchiolar metaplasia, fibroblast activation, and extracellular matrix (ECM) deposition. Aberrant epithelial remodelling is associated with the release of tumour-associated epithelial biomarkers, including CA19-9, CA125, and CYFRA21-1, which can be detected in the peripheral circulation.

**Table 2:** Biomarker-centered summary of the available evidence, assessment strategy and kinetics, potential clinical relevance, and main limitations of circulating biomarkers investigated in ANCA-associated vasculitis-associated interstitial lung disease (AAV-ILD). Potential clinical relevance is based on currently available observational evidence and should be considered hypothesis-generating rather than a validated indication for routine clinical practice. None of the biomarkers summarized in this table has yet undergone sufficient prospective external validation to support biomarker-guided management in routine AAV-ILD care.

Biomarker	Main Evidence/Investigated Role	Assessment and Kinetics	Potential Clinical Relevance	Main Limitations
KL-6 [12,13,14,15]	Higher concentrations reported in AAV-ILD; associated with pulmonary involvement and, in REVEAL, with subsequent ILD relapse	Baseline assessment with limited serial data; changes during the clinical course reported in small cohorts	Candidate marker for pulmonary involvement, longitudinal assessment, and relapse-risk stratification	Small diagnostic cohorts; very limited standardized serial evidence; heterogeneous thresholds; not AAV-specific; no external validation
SP-D [14,15]	Higher in the clinically fibrotic-dominant MPA-ILD phenotype; independently associated with subsequent ILD relapse in REVEAL	Predominantly baseline assessment; post-treatment serial kinetics not established	Candidate marker for fibrotic-phenotype characterization and relapse-risk stratification	Few AAV-ILD studies; limited measurements; cohort-derived threshold; no external validation
CCL2/MCP-1 [15,16]	Higher in MPA-ILD; higher baseline levels associated with subsequent increase in HRCT fibrosis score	Baseline and 1-year measurements available; serial increase did not correlate with change in fibrosis score	Candidate marker for MPA-ILD association and future fibrotic evolution	Small single-centre cohort; systemic/non-lung-specific chemokine; no external validation
CXCL13 [15]	Baseline levels correlated with HRCT ground-glass opacity and fibrosis scores	Single pretreatment measurement	Exploratory marker of radiological inflammatory/fibrotic phenotype	Single small cohort; correlation-based evidence; no clinical threshold or external validation
IL-13 [15]	Baseline levels correlated with HRCT fibrosis score	Single pretreatment measurement	Exploratory marker of Th2-associated fibrotic phenotype	Single small cohort; modest correlation; systemic/non-lung-specific cytokine; no threshold or external validation
CXCL6 [17]	Higher in AAV-ILD; high baseline levels independently associated with subsequent ILD progression	Single baseline measurement followed by longitudinal clinical assessment; serial CXCL6 kinetics not evaluated	Candidate baseline marker for pulmonary involvement and progression-risk stratification	Single-centre evidence; correlation with systemic inflammation limits pulmonary specificity; no independent validation
CA19-9 [18]	Higher in MPO-AAV-ILD; associated with ILD, HRCT fibrosis burden, and all-cause mortality	Baseline assessment; serial kinetics not established	Candidate adjunct marker for ILD association, fibrotic burden, and prognostic investigation	Single retrospective cohort; low sensitivity despite high specificity; limited disease/organ specificity; no external validation
CA125 [18]	Higher in MPO-AAV-ILD	Baseline assessment; serial kinetics not established	Exploratory adjunct marker of pulmonary involvement and disease severity	Single retrospective cohort; modest discriminatory performance; limited organ specificity; no externally validated threshold
CYFRA21-1 [18]	Higher in MPO-AAV-ILD; associated with HRCT ground-glass opacity and all-cause mortality	Baseline assessment; serial kinetics not established	Candidate adjunct marker for radiological and prognostic investigation	Single retrospective cohort; moderate discrimination; limited organ specificity; potentially influenced by renal dysfunction; no external validation

## Data Availability

The datasets generated during and/or analyzed during the current study are available from the corresponding author on reasonable request.

## Supplementary Materials

The following supporting information can be downloaded at: https://www.mdpi.com/article/10.3390/biomedicines14102202/s1, Table S1: Complete database-specific search strategies used to identify studies investigating circulating biomarkers in ANCA-associated vasculitis-associated interstitial lung disease.

## Abbreviations

The following abbreviations are used in this manuscript:

ILD	Interstitial lung disease
AAV	ANCA-associated vasculitis
AAV-ILD	AAV-associated ILD
BVAS	Birmingham Vasculitis Activity Score
CCL-2	C-C motif chemokine ligand 2
CXCL-6	C-X-C motif chemokine ligand 6
CA19-9	Carbohydrate antigen 19-9
CA-125	Cancer antigen 125
CYFRA21-1	Cytokeratin-19 fragment
DAH	Diffuse alveolar hemorrhage
KL-6	Krebs von den Lungen-6
EGPA	Eosinophilic granulomatosis with polyangiitis
GPA	Granulomatosis with polyangiitis
HRCT	High-resolution computed tomography
MPA	Microscopic polyangiitis
MPO	Myeloperoxidase
SP-D	Surfactant protein D
UIP	Usual interstitial pneumonia
NSIP	Nonspecific interstitial pneumonia
OP	Organizing pneumonia
